# Periderm invasion contributes to epithelial formation in the teleost pharynx

**DOI:** 10.1038/s41598-019-46040-y

**Published:** 2019-07-12

**Authors:** Joana Teixeira Rosa, Veronika Oralová, Daria Larionova, G. T. Eisenhoffer, P. Eckhard Witten, Ann Huysseune

**Affiliations:** 1 Research Group Evolutionary Developmental Biology, Biology Department, Ghent University, K.L. Ledeganckstraat 35, B-9000 Gent, Belgium; 2 Present Address: Comparative, adaptive and functional skeletal biology (BIOSKEL), Centre of Marine Sciences (CCMAR), Building 7, University of Algarve, Campus Gambelas, 8005-139 Faro, Portugal; 3 Present Address: Institute of Animal Physiology and Genetics, v.v.i., Czech Academy of Sciences, Veveri 97, 602 00 Brno, Czech Republic; 4 Department of Genetics, The University of Texas MD Anderson Cancer Center, 1515 Holcombe Blvd, Unit 1010, Houston, Texas 77030 USA

**Keywords:** Developmental biology, Evolution

## Abstract

The gnathostome pharyngeal cavity functions in food transport and respiration. In amniotes the mouth and nares are the only channels allowing direct contact between internal and external epithelia. In teleost fish, gill slits arise through opening of endodermal pouches and connect the pharynx to the exterior. Using transgenic zebrafish lines, cell tracing, live imaging and different markers, we investigated if pharyngeal openings enable epithelial invasion and how this modifies the pharyngeal epithelium. We conclude that in zebrafish the pharyngeal endoderm becomes overlain by cells with a peridermal phenotype. In a wave starting from pouch 2, peridermal cells from the outer skin layer invade the successive pouches until halfway their depth. Here the peridermal cells connect to a population of cells inside the pharyngeal cavity that express periderm markers, yet do not invade from outside. The latter population expands along the midline from anterior to posterior until the esophagus-gut boundary. Together, our results show a novel role for the periderm as an internal epithelium becomes adapted to function as an external surface.

## Introduction

Anatomically, the vertebrate pharynx corresponds to the part of the digestive tube that is surrounded by the post-mandibular visceral arches^1^. In teleosts, the most speciose group of vertebrates, the pharynx functions in food transport and manipulation. For respiration, water taken up via the mouth passes over the internal gills and is expelled via the gill slits. Thus, different from amniotes, the pharynx has channels allowing direct communication between internal and external environment. Yet, as in amniotes, the pharynx is lined by endoderm, an epithelium typically paving the (internal) alimentary tube^2^. Whether the teleost pharyngeal epithelium is modified during early development to prepare for direct interaction with the external environment is not known. In particular, if opening of the endodermal pouches (prospective gill slits) affects the composition of the pharynx lining has not been properly investigated.

Apart from its function in feeding and as a respiratory channel, the pharynx lining forms the anlagen of widely divergent organs such as lungs, gills, or teeth, and an array of endocrine organs, such as thyroid glands, ultimobranchial bodies, and the thymus (reviewed in^3,4^). Moreover, signaling from the pharyngeal epithelium is required for proper development of the visceral skeleton (e.g.^5,6^; reviewed in^7,8^). With respect to teleosts and other primary aquatic gnathostomes, the question raises if the original endoderm fulfills these functions. Possibly, because of the early contact with the external environment the epithelium must first undergo secondary modifications, such as stratification. The origin of the cell layers that contributes to the pharyngeal epithelium has captured the interest of generations of researchers. Discussions focused on how far ectoderm invades the oral cavity via the mouth in different vertebrate lineages (e.g.^9,10^) and how much it contributes to organs derived from the oropharyngeal lining, such as gills^11–13^ or taste buds^14^. Little attention has been paid to the pouches as potential passageways for ectoderm to invade the pharynx and to potentially contribute to pharyngeal derivatives.

Here we examine how the zebrafish pharyngeal epithelium becomes modified in early development. We test if, and how far, cells of the outer body covering (ectoderm and/or periderm) enter the pharynx through the pharyngeal pouches and gill slits. We also examine if and how cells in the pharynx midline change during early development.

## Results

### Peridermal cells enter pouches 2 to 6

**Figure 1:**
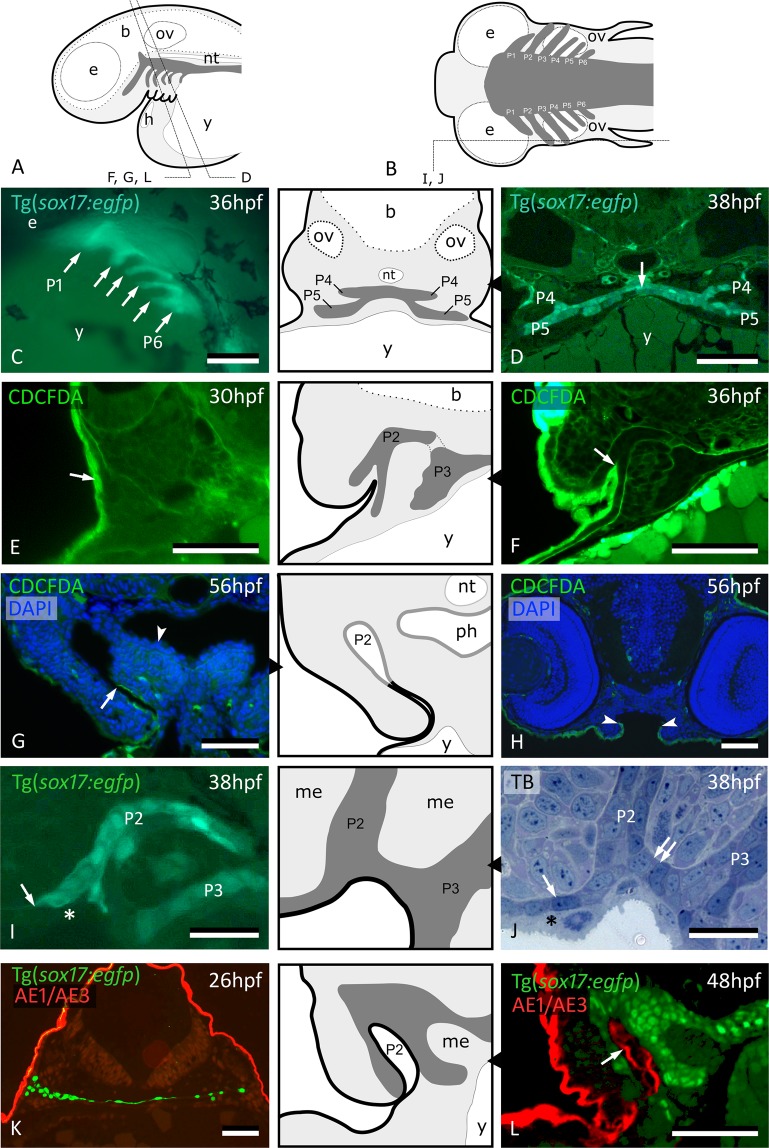
Peridermal cells invade pouch 2. (**A**,**B**) Generalized scheme of zebrafish pouches (dark grey) in a lateral (A) and horizontal (B) view, with sections D-L as indicated. (**C**) Lateral view of Tg(*sox17:egfp*) embryo showing pouches 1 to 6 (compare to (A)). (**D**) GFP labeled endodermal cells along the midline (white arrow) extend into pouches 4 and 5 (P4 and P5, resp.); corresponding schematic attached. (**E**) CDCFDA labeling at 26 hpf, sacrifice at 30 hpf; labeled cells are exclusively located in the superficial skin layer or periderm (arrow, level of pouch 2). (**F**) CDCFDA labeling at 26 hpf, sacrifice at 36 hpf; peridermal cells have partially invaded pouch 2 (arrow); corresponding schematic attached. (**G**) CDCFDA labeling at 26 hpf, sacrifice at 56 hpf; peridermal cells (arrow) line the now open gill slit; elsewhere the pharyngeal epithelium remains free of peridermal cells (arrowhead); corresponding schematic attached. (**H**) CDCFDA labeled cells only cover part of the epithelial lining of the mouth. Their boundary is indicated by arrowheads and coincides with the reduction of two to one epithelial cell layer. (**I**,**J**) Contact area of pouches 2 and 3 with skin; note that *sox17*+ cells spread out (arrows) and appear to displace the basal cell layer. In this way, the distal parts of the pouches even become connected (double arrow). Only periderm (asterisks) covers the expansion of *sox17*+ endodermal cells. Corresponding schematic attached. (**K**,**L**) Immunolabeling using the pan-cytokeratin marker AE1/AE3 on Tg(*sox17:egfp*) embryos labels periderm only at 26 hpf (**K**), but also marks the lining of P2 at 48 hpf (arrow) (**L**); corresponding schematic attached. In all schemes, endoderm is dark gey, periderm is a black line. (**C**) Whole mount embryo; (**D**–**H**,**K**,**L**) cross sections; (**I**,**J**) sagittal sections. b: brain; e: eye; h: heart; me: mesenchyme; nt: notochord; ov: otic vesicle; P2 – P6: pouches 2 to 6; ph: pharyngeal lumen; TB: toluidine blue staining; y: yolk. Scale bar (**C**) = 500 μm, (**D**–**H**,**K**,**L**) = 50 μm, (**I**,**J**) = 20 µm.

The pharyngeal endoderm becomes distinct from the mesoderm at about 10^1/4^ hours post fertilization (hpf)^15^. Using a transgenic line (Tg(*sox*17*:egfp*)) that marks the endodermal cells, pharyngeal pouches can be observed to develop sequentially from anterior (pouch 1, P1) to posterior (pouch 6, P6) between 18 hpf and 38hpf (Fig. 1A–C), confirming earlier reports^16–18^. Initially, the endoderm constitutes a single layer of flattened cells along the midline of the body, overlying the yolk. Soon after, this monolayer expands into two apposed layers of *sox17*+ cells (Fig. 1D). Each pouch is constituted of two simple closely apposed *sox17*+ epithelia, extending pairwise on each side of the midline endoderm and reaching up to the body surface (Fig. 1B,D). Because of the intimate contact between pouch endoderm and body surface, presaging the opening of the gill slit, we investigated if, when and how cells from the embryonic skin (either the superficial, periderm layer or the basal, ectodermal layer) colonize the pouches and the midline endoderm. To do so, we monitored cell dynamics using [5-(and-6)-carboxy-2’,7’-dichlorofluorescein diacetate, succinimidyl ester, mixed isomers] (CDCFDA) as a cell tracer and investigated the distribution of ectoderm/and or periderm using different markers.

Labeling of wildtype (AB line) embryos using CDCFDA for 4 hrs, starting at 26 hpf, exclusively marked the outer, i.e., peridermal, skin layer (Fig. 1E). Labeled peridermal cells start to appear in pouch 2 (P2) at around 32 hpf and extend inwards until about halfway the pouch (Fig. 1F), but never reach the midline endoderm, not even as late as at 56 hpf (Fig. 1G). Peridermal cells also expand into the stomodeum. Similar to the invasion of P2 (and other pharyngeal pouches, see below), migration of CDCFDA-labeled cells in the forming mouth is arrested. Labeled cells do not cover the roof of the future mouth (Fig. 1H). The limit of CDCFDA labeled cells coincides with a reduction from two to one epithelial cell layers resting on the basal lamina. The basal ectodermal cells are not observed at any time within P2. Rather, they appear to be pushed away from the prospective gill slits by *sox17*+ cells from the expanding distal tip of the pouch (Fig. 1I,J).

Immunolabeling with the pan-cytokeratin AE1/AE3 antibody at 26 hpf marked periderm only (Fig. 1K). In contrast, immunostaining at 48 hpf showed labeled cells extending halfway P2 (Fig. 1L, compare to 1G), confirming the results obtained by cell tracing.

**Figure 2:**
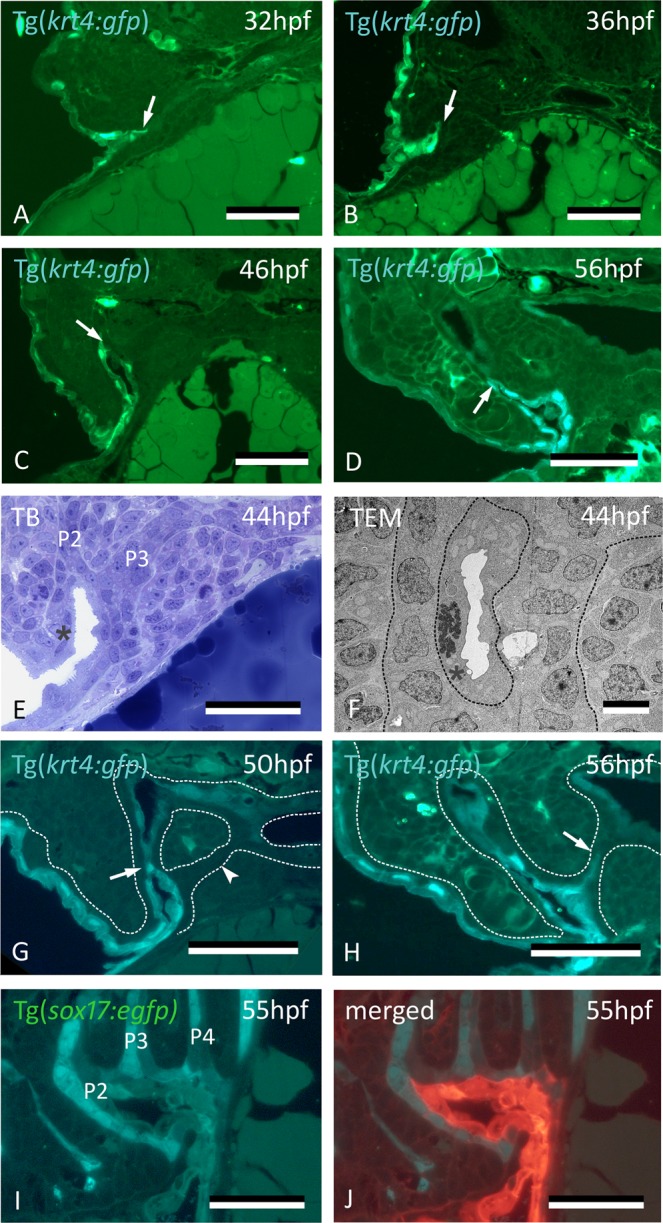
Peridermal cells sequentially invade pouches 2 to 6 but do not reach the midline endoderm. (**A**–**C**) *krt4*+ cells are present in progressively deeper parts of pouch 2 (P2) (arrows). (**D**) Peridermal cells are arrested approximately halfway the pouch (arrow). (**E**,**F**) At 44 hpf P2 is open to the exterior. Peridermal cells extend inside the pouch (black stars); a lumen forms only where these cells cover the endodermal layer. In (**F**), outer dotted lines mark contour of the pouch; inner dotted line marks contour of peridermal cells. (**G**) While peridermal cells have entered through P2 (arrow), *krt4*+ cells remain excluded from P3 (arrowhead) and more posterior pouches. (**H**) At 56 hpf, peridermal cells have started to enter P3 (arrow), and progressively extend inwards. (**I**,**J**) Double transgenics Tg(*sox17:egfp;krt4:tomato*) show opening of P2 with periderm entering and covering *sox17*+ endodermal cells (I, green channel only; J, overlay). P2–P6: pouches 2 to 6. (**A**–**H**) cross sections, (**I**,**J**) sagittal sections. TB: toluidine blue staining. Scale bars (**A**–**E**) and (**G**–**J**) = 50 μm, (**F**) = 5 μm.

The fate of the cells labeled by CDCFDA was further confirmed using the Tg(*krt4:gfp*) line^19^. Keratin 4-positive (*krt4*+) peridermal cells start to appear in the distal part of pouch 2 (P2) at around 32 hpf (Fig. 2A), extending into the pouch for about halfway of its length (Fig. 2B). Cells squeeze between the two apposed layers of endoderm, thus separating them and paving each single layer of endoderm with a layer of *krt4*+ cells. Lumen formation in P2 starts at 36 hpf, and typically occurs where peridermal cells have covered the endoderm (Fig. 2C–F). By 60 hpf, the pouch has completely opened, creating the first open gill slit (Suppl. Fig. S1). The opercular flap is now a distinct structure, its surface completely covered with labeled cells. Following the invasion of P2, but considerably later, *krt4*+ peridermal cells sequentially enter P3-P6 from the outside (Fig. 2G,H). Invasion occurs at 56 hpf for P3 and P4 and at 72 hpf for P5 and P6, respectively (Suppl. Fig. S5). As in P2, invasion is arrested about halfway in each pouch (Fig. 2H). Double transgenic embryos revealing both endoderm and periderm (Tg(*sox17:egfp*; *krt4:tomatoCAAX*)) confirmed that, by 55 hpf, periderm has partially invaded P2 and covers *sox17*-positive endodermal cells (Fig. 2I,J).

### *krt4*+ cells expand through the midline posteriorly to cover the entire endodermal lining

**Figure 3:**
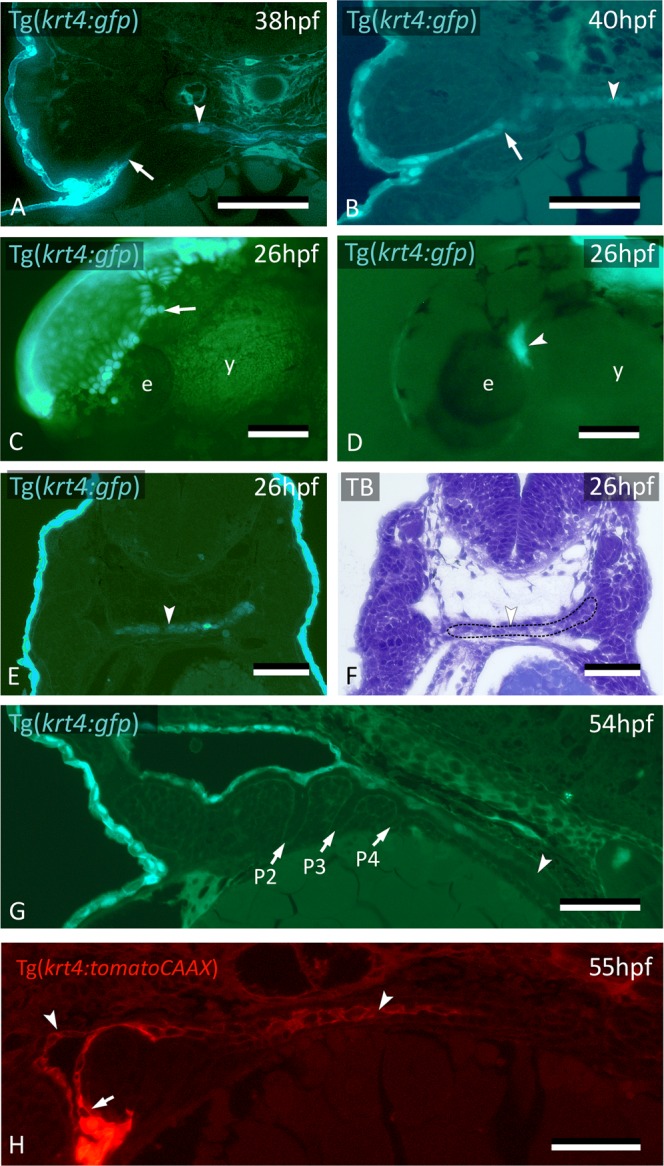
*krt4*+ cells expand along the midline and connect to invaded peridermal cells. (**A**) At 38 hpf, *krt4*+ cells are visible along the midline (arrowhead), separated from invading peridermal cells (arrow). (**B**) Shortly after, the midline *krt4*+ cells (arrowhead) make contact with peridermal cells that have entered through P2 (arrow). (**C**) Lateral view on Tg(*krt4:gfp*) embryo in the early phase of periderm removal with EDTA. Part of the periderm is still attached (arrow). (**D**) At an advanced stage of EDTA treatment, the periderm is completely removed, and an internal *krt4*+ structure has become visible (arrowhead). (**E**,**F**) This internal *krt4*+ structure (arrowhead on E) coincides with the midline endoderm at the level of pouch 1–2 (arrowhead on F). (**G**) By 54 hpf, midline *krt4*+ cells cover the entire pharynx lining (boundary indicated by arrowhead). P2 is wide open (at another level of sectioning) but other pouches (P3–P6) are still free of *krt4*+ cells. (**H**) Double transgenic embryo Tg(*sox17:egfp;krt4:tomato*) of 55 hpf shows lining of pharyngeal epithelium with *krt4*+ cells (arrowheads) (red channel only). Note continuity, albeit with sharp boundary (arrow), with peridermal cells entering via P2. (**A**,**B**,**E**,**F**) Cross sections; (**C**,**D**) whole mount embryos; (**G**,**H**) sagittal section. TB: toluidine blue staining. e: eye; y: yolk; P2–P6: pouches 2 to 6. Scale bars (**A**,**B**,**E–G**) = 50 μm; (**C**,**D**) **=** 100 μm; (H) = 20 µm.

Surprisingly, cells expressing the peridermal marker (*krt4*+) were also observed along the midline, trapped between the two endodermal layers (Fig. 3A,B). Because cell lineage tracing had clearly shown that peridermal cells from the skin do not reach the midline, these *krt4*+ cells (further called midline *krt4*+ cells) must have another origin.

First, we confirmed the independent origin of these midline *krt4*+ cells from the periderm by removing the periderm with EDTA from Tg(*krt4:gfp*) embryos aged 26 hpf, i.e. well before peridermal cells start to invade P2 (Fig. 3C). This experiment revealed an internal cluster of cells showing marked *krt4* expression present at this time point (Fig. 3D). To investigate if the cluster of midline *krt4*+ cells derives from periderm at an earlier stage of development, we labeled embryos with CDCFDA for 4 hrs starting at 6 hpf, every two hours until 22 hpf. In none of these specimens did we ever observe periderm entering that could explain the cluster of *krt4*+ cells observed along the midline at the level of P1-P2 (data not shown).

At 26 hpf, the midline *krt4*+ cells are first located anteriorly, in a region potentially coinciding with endoderm (Fig. 3E,F). The midline *krt4*+ cells are initially separated from the peridermal cells invading P2 but contact them later (Fig. 3A,B, Supplementary Fig. S1). The domain of midline *krt4*-expressing cells is then seen to expand from the region of P2 posteriorly along the midline between the two layers of endodermal cells, reaching pouch 3 (P3), P4, P5 and P6 at 38 hpf, 42 hpf, 46 hpf and 48 hpf, respectively (Supplementary Fig. S2). At 54 hpf, the entire pharyngeal lumen, as well as P2, is covered by *krt4* expressing cells (Fig. 3G). This was confirmed by Tg(*sox17:egfp*; *krt4:tomatoCAAX*) embryos (Fig. 3H). The appearance of *krt4*+ cells in the pharynx midline anteriorly, and their expansion into the posterior pharynx, was also confirmed through time lapse imaging of Tg(*krt4:gfp*) embryos using dual photon laser scanning microscopy (Supplementary Fig. S3 and Supplementary Movie).

**Figure 4:**
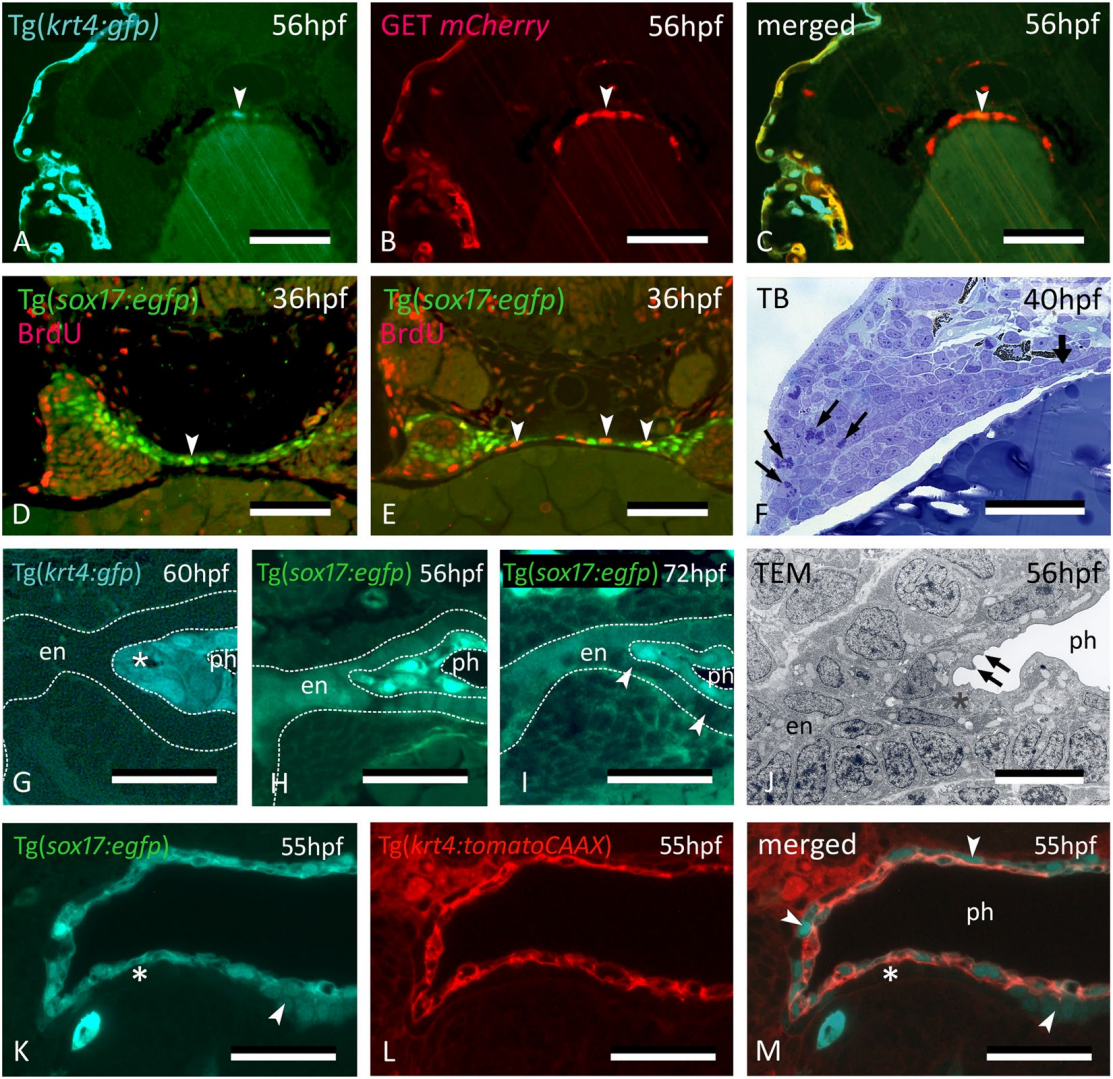
Midline *krt4*+ cells are distinct from peridermal and endodermal cells. (**A–C**) In embryos of GET-periderm x Tg(*krt4:gfp*) line, at 56 hpf, both the periderm and the midline *krt4*+ cells (arrowhead) strongly express mCherry in patterns overlapping with the *krt4:GFP* + cells. (**D**,**E**) Mitoses (arrowheads) revealed by BrdU pulse labeling at 36 hpf in the midline endoderm at more anterior (**D**) and posterior (**E**) cross sectional level. (**F**) Abundant mitoses in the distal part of the pouch (arrows) but not in the midline endoderm (thick arrow). (**G**) Midline cells (star) maintain a strong *krt4*+ expression at 60 hpf. (**H**,**I**) Downregulation of *sox17*+ expression in the endodermal layer (delimited by the outer dotted line in H, and by arrowheads in I) but upregulation in the superficial layer surrounding the pharyngeal lumen (delimited by inner two dotted lines). (**J**) Cells of the superficial layer are flattened and more electron-dense in TEM than the basal endodermal layer, composed of cuboidal cells, and they develop microridges (arrows). Epidermis to the left; midline to the right. (**K–M**) Double transgenic embryo Tg(*sox17:egfp;krt4:tomato*) of 55 hpf; *krt4*+ cells are positive for *sox17*; conversely, only some cells of the basal endodermal layer have retained a *sox17* signal (arrowheads). (**A–M**) cross sections. en: endodermal layer; ph: pharyngeal lumen; TB: toluidine blue staining. Scale bars (**A–F**) **=** 50 μm, (**G**–**I**) = 25 µm, (**J–M**) = 10 µm.

The *krt4*+ and thus periderm-like phenotype of the midline cells was further corroborated by using embryos of the Et(Gal4-VP16)^zc1044A^;Tg(UAS-E1b:nsfB-mCherry)^c264^ line, validated as having expression in the periderm^20^, crossed to the Tg(*krt4:gfp*) line. In embryos sacrificed at 56 hpf, both the periderm and the midline *krt4*+ cells strongly express mCherry in patterns overlapping with the *krt4:GFP* + cells, indicating that the same enhancer is active in both cell types, peridermal cells and midline cells (Fig. 4A–C).

On the other hand, staining of Tg(*sox17:egfp*) embryos with the pan-cytokeratin antibody AE1/AE3 yielded a strong signal in the periderm but did not label midline *krt4*+ cells (Fig. 1K,L). This indicates that not all keratins are shared by both cell populations. Zn-8, which detects Alcama protein and is an established endodermal pouch marker, labels pouches but not the midline endoderm. Hence, it could not be used to perform a reciprocal colocalization experiment (staining Tg(*krt4:gfp*) embryos with an endoderm marker).

To examine whether mitoses contribute to stratification of the pharynx along the midline, we assessed proliferation on semithin sections and by 5-bromo-2′-deoxyuridine (BrdU) labeling (Fig. 4D–F). Using a short pulse at 36 hpf, proliferating cells were observed along the midline, albeit more frequent where the endoderm had not yet stratified into two opposing monolayers (compare Fig. 4D,E). This suggests that mitoses contribute to establish the endoderm bilayer, but not (or little) to the *krt4*+ cells that cover them. Proliferation was abundant in the pouches proper (Fig. 4F).

The midline cells maintain *krt4* expression for an extended period (Fig. 4G), and even at 5 dpf, the signal is still very strong. Remarkably, while the basal endodermal cells turn off *sox17* expression at around 72 hpf, the midline *krt4*+ cells that cover them start expressing *sox17* strongly at around 56 hpf (Fig. 4H,I). The ultrastructure of the two cell types also differs (Fig. 4J). Superficial cells (corresponding to the *krt4*+ cells) develop microridges, are more squamous, with a flattened nucleus, and possess a more electron-dense cytoplasm than the underlying endodermal cells. Double transgenic embryos (Tg(*sox17:egfp*; *krt4:tomatoCAAX*) confirmed overlapping *krt4* and *sox17* expression in the superficial layer, as opposed to exclusive, yet fading, *sox17* expression in the basal endodermal layer (Fig. 4K). Again, as with P2, a lumen forms only when and where *krt4*+ cells have covered the endoderm (Fig. 4K–M).

### Midline *krt4*+ cells expand up to the gut entrance and join peridermal cells invaded through pouches 3–6

**Figure 5:**
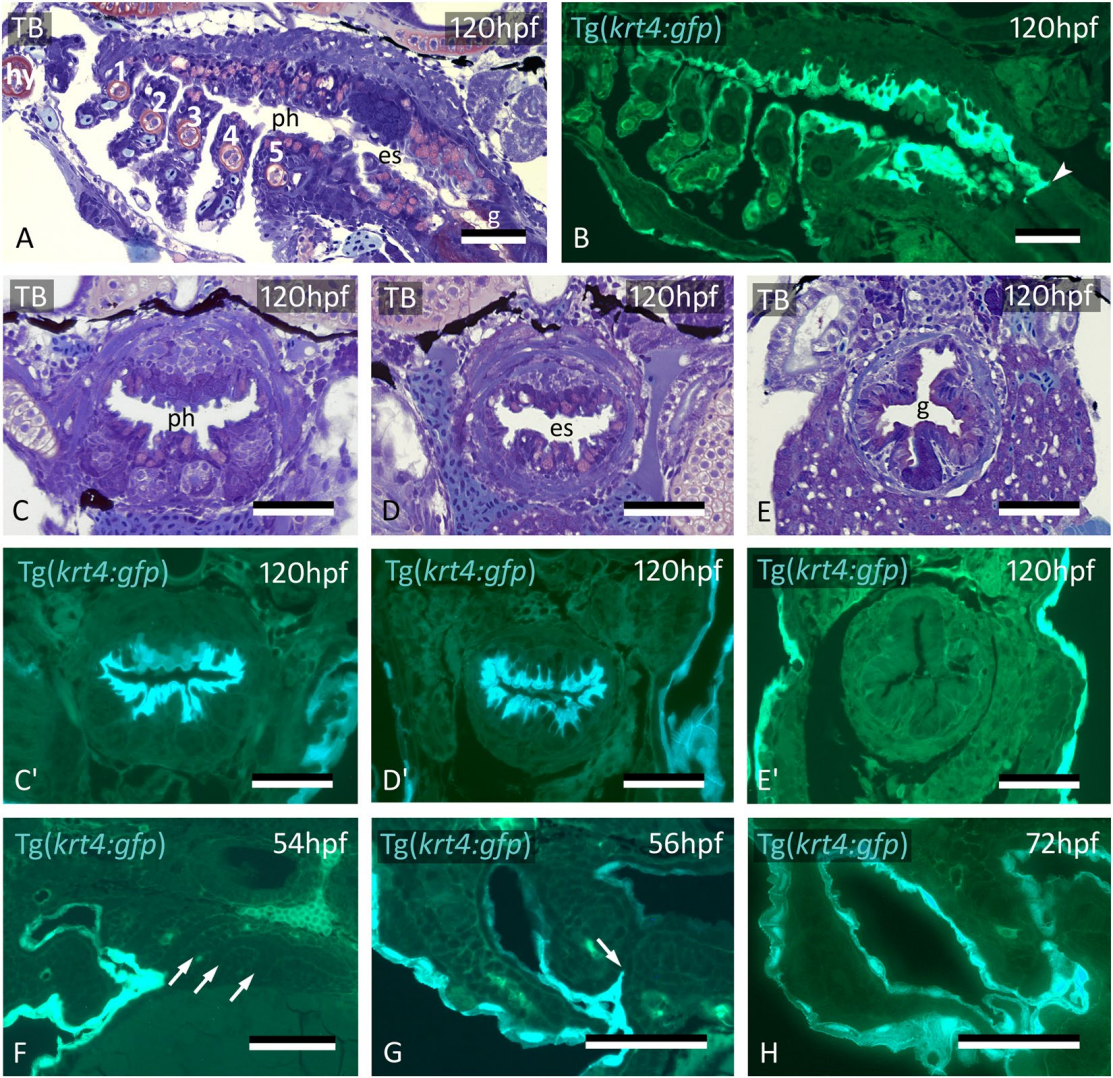
Midline *krt4*+ cells cover pharynx and esophagus, and meet peridermal cells invading through P3-P6. (**A**,**B**) *krt4*+ cells cover hyoid arch (hy) and branchial arches 1 to 5 (numbered 1–5), as well as roof and floor of the pharynx and esophagus. Note sharp boundary (arrowhead) between *krt4*+ esophagus and *krt4*- lining of the gut. (**C**,**C’**,**D**,**D’**) *krt4*+ cells cover the lining of the pharynx (**C**,**C’**) and the esophagus (**D**,**D’**), resp. Note long cell extensions issuing from the *krt4*+ cells (**E**,**E’**) Absence of *krt4*+ cells in the gut coincides with single-layered epithelium. (**F**) At 54 hpf, pouches 3–6 are still free of *krt4*+ cells. (**G**,**H**) Progressive invasion of P3 (arrow) by peridermal cells that meet midline *krt4*+ cells. (**A**,**B**,**F**) sagittal sections, (**C–E’**,**G**,**H**) cross sections. es: esophagus; g: gut; ph: pharyngeal lumen; TB: toluidine blue staining. Scale bars (**A–H**) **=** 50 μm.

The domain of *krt4*+ cells has a sharp posterior limit at the end of the esophagus (Fig. 5A,B). The boundary coincides with the transition from a bilayered to a single-layered epithelium that lines the gut (Fig. 5C–E’). The single-layered gut epithelium is *krt4*-negative (Fig. 5E’). Where the epithelial lining is bilayered, the superficial *krt4*+ cells display long cell extensions that reach down to the basal lamina in-between the endodermal cells (Fig. 5C’,D’).

During invasion of P2 by peridermal *krt4*+ cells, and during expansion of midline *krt4*+ cells towards the posterior pharynx, the other pouches persist as two apposed simple endodermal epithelia, free of *krt4*+ cells (Fig. 5F, Supplementary Fig. S2, S4). Midline *krt4*+ cells, present before peridermal cells enter P3 to P6, extend outwards through pouches 3–6. Eventually, they join the *krt4*+ cells that enter these pouches from outside, between 56 and 72 hpf (Fig. 5G,H), in a way similar as observed for P2 (compare with Fig. 3B; Supplementary Fig. S5). Thus, the simple endodermal epithelium resting on the basal lamina in the midline, as well as in each pouch, becomes completely overlain by an uninterrupted layer of *krt4*+ cells. This double lining is completed at 72 hpf for P3 and P4 and at 84 hpf for P5 and P6 (Supplementary Fig. S5, S6). Again, a lumen appears in the pouch only after it is invested with *krt4*+ cells. After the midline *krt4*+ cells that extend outwards in each pouch have joined with the peridermal *krt4*+ cells extending inwards, a boundary cannot be observed anymore between both *gfp* + cell populations, although it remains visible between *krt4:tomatoCAAX* + periderm and midline cells in double transgenic (Tg(*sox17:egfp*; *krt4:tomatoCAAX*) embryos (Fig. 3H). Thus, the entire pharyngeal cavity as well as the opened gill slits at 56 hpf and beyond are lined by a continuous *krt4*+ layer, covering the original endodermal layer.

### Cells resembling periderm cells line the pharynx in other teleost species

**Figure 6:**
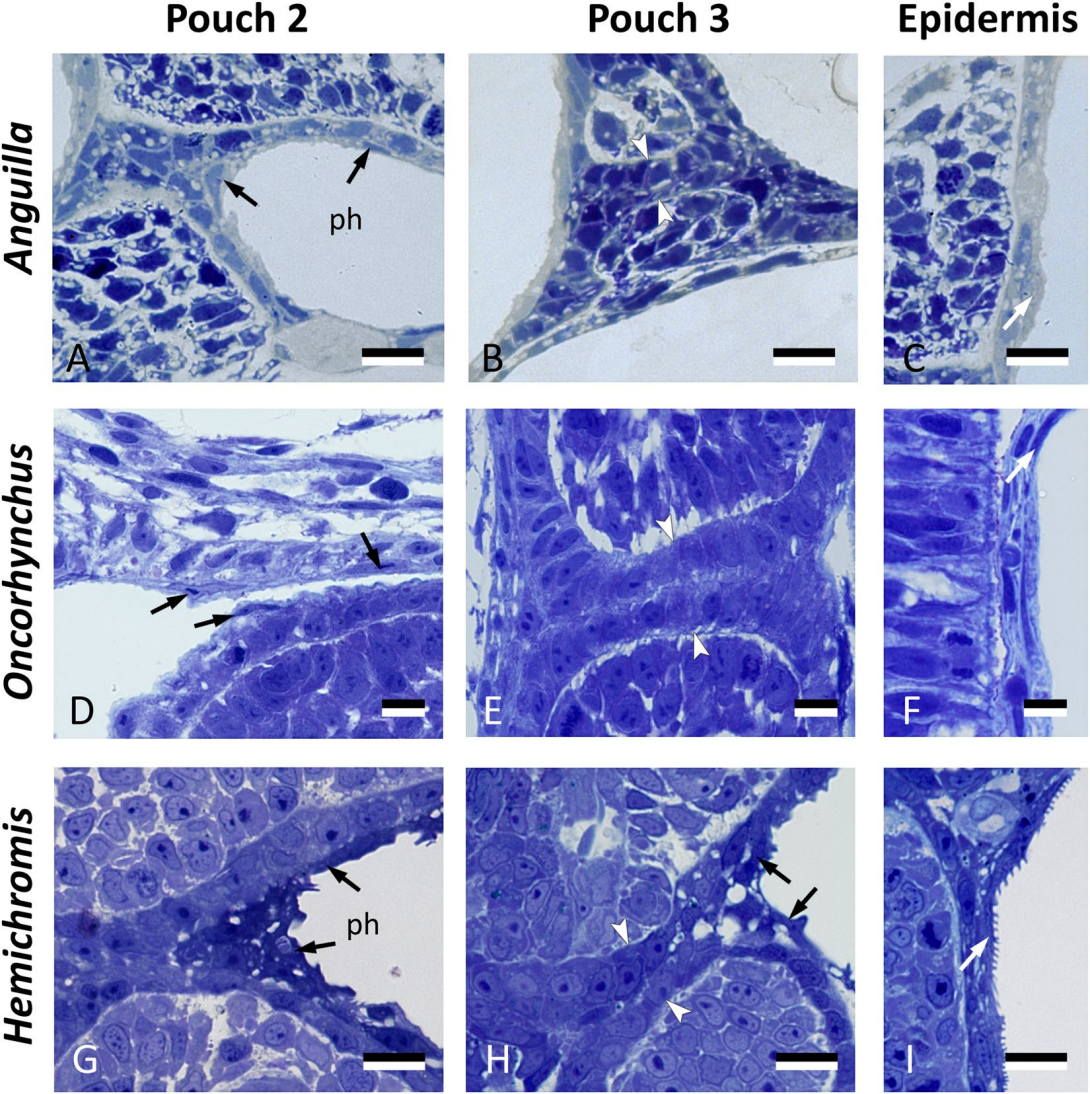
Cells resembling periderm cells in the pharynx of other teleost species. Cross sections through the region of pouch 2, pouch 3 and comparison to the skin in three teleost species: *Anguilla anguilla* (total length, TL, 6.0 mm), *Oncorhynchus tshawytscha* (unhatched embryo, 15 dpf), and *Hemichromis bimaculatus* (TL 4.0 mm, 1 day post-hatching, dph). Pouch 2 (**A**,**D**,**G**) is lined by flattened cells (black arrows) overlying a layer of cuboidal cells. Where pouch 3 is not open yet (**B**,**E**), only a double layer of cuboidal endodermal cells is present (between white arrowheads). Where pouch 3 starts to open (**H**), similar cells can be seen to cover the pouch endoderm (black arrows). Note resemblance, for each of the three species, to peridermal cells in the superficial skin cover (white arrows, **C**,**F**,**I**). In (**A**,**B**,**D**,**E**,**G**,**H**), the epidermis is to the left and the pharyngeal lumen to the right; in (**C**,**F**,**I**) the external surface is to the right. (**A–I**) cross sections. ph: pharyngeal lumen. Scale bars (**A–I**) **=** 10 μm.

To investigate if a layer of flattened cells with periderm-like phenotype covers the pharyngeal endoderm in other teleosts, we did a preliminary survey of three widely divergent teleost species: European eel (*Anguilla anguilla*, Anguilliformes, a basal teleost species), Pacific salmon (*Oncorhynchus tshawytscha*, Salmoniformes), and the Jewel cichlid (*Hemichromis bimaculatus*, Perciformes, a highly derived teleost species). In the absence of markers or transgenic lines for these species, we compared the histology of the skin with the region of pouches 2–3 in early developmental stages (Fig. 6). In all three species, pouch 2, and the opened portion of pouch 3, appeared to be lined by flattened cells, resembling peridermal cells in the superficial skin cover, and overlying a layer of cuboidal cells, inferred to be the endoderm. Where pouch 3 was not open yet, only a double layer of cuboidal endodermal cells was observed.

## Discussion

**Figure 7:**
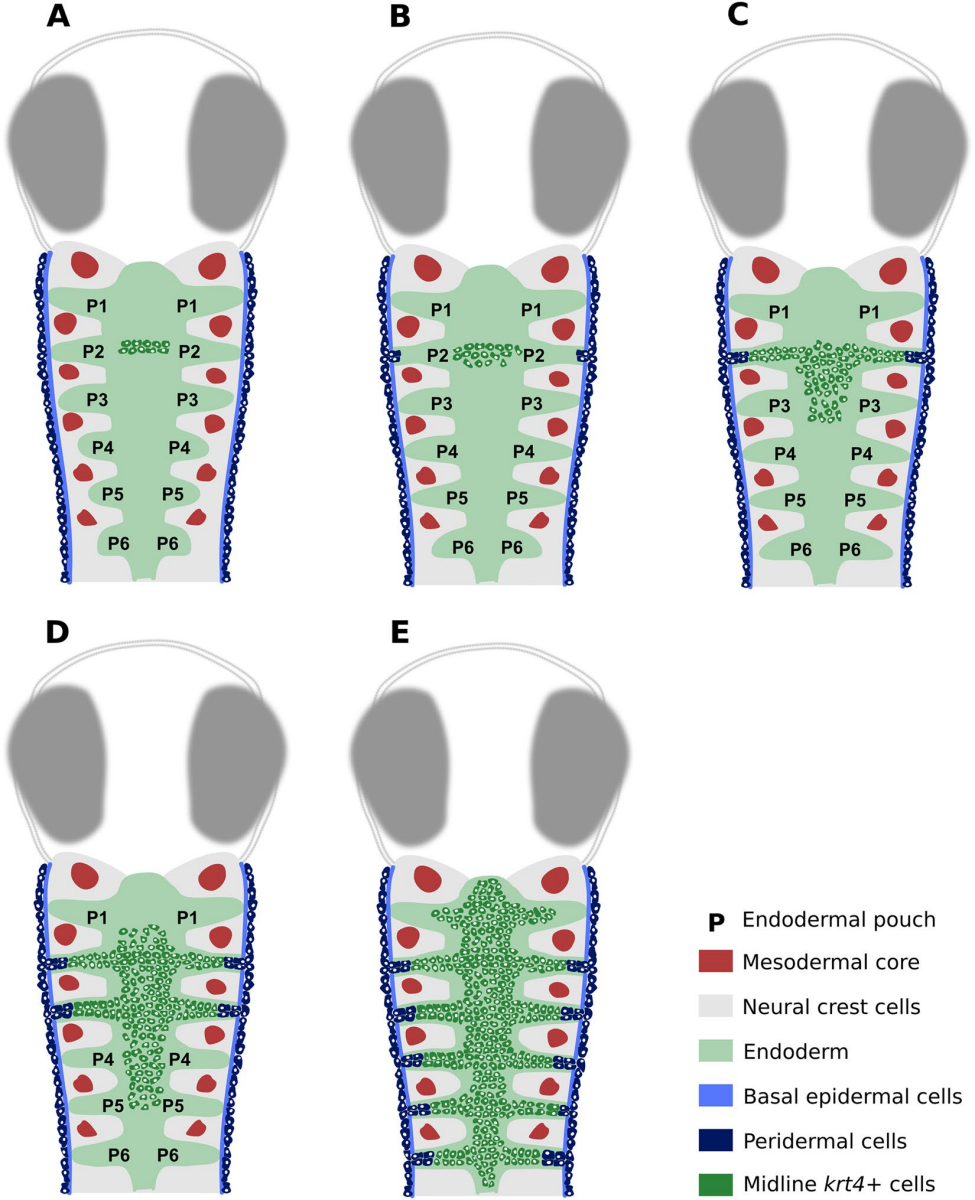
Interpretative scheme, showing development of the pharyngeal lining. (**A**) Before 30 hpf, peridermal (*krt4*+) cells are only seen covering the embryo; internal *krt4*+ cells are only present in the midline at the level of P1-P2. (**B**) Around 32 hpf peridermal cells start to invade P2. The domain of midline *krt4*+ cells starts to spread posteriorly and laterally towards P2. (**C**) Peridermal cells invade P2 until halfway and midline *krt4*+ cells extend outwards reaching peridermal *krt4*+ cells. Also midline *krt4*+ cells continue to extend through the pharynx midline until the level of P3. (**D**) The invasion and expansion of both peridermal and midline *krt4*+ cells continue, following the same pattern seen for P2. (**E**) Finally, the entire pharynx midline is invested by *krt4*+ cells and all the pouches, until halfway, are covered in peridermal cells. The domain of midline *krt4*+ cells also expands anteriorly until the mouth, and posteriorly until the end of the esophagus. P2: pouch 2; P3: pouch 3; P4: pouch 4; P5: pouch 5; P6: pouch 6.

We show here that the pharynx and the gill slits in early post-embryonic zebrafish are lined by a double-layered epithelium: a basal layer resting on the basal lamina, consisting of *sox17*+ endoderm, and a superficial *krt4*+ layer, derived partly from the skin periderm (in the pouches), and partly from internal anteriorly located cells that start to express *krt4*. Lineage tracing experiments along with observations on transgenic embryos clearly reveal that peridermal cells enter all pouches, in succession from anterior (P2) to posterior (P6), albeit not up to the midline. Instead, the cells are met by *krt4*+ cells, whose domain expands along the midline, likewise from anterior to posterior (Fig. 7). The midline cells turn on *krt4* expression before the start of invasion of *krt4*+ peridermal cells through pouch 2. Our observations confirm the dynamic pattern of *krt4* expression observed by *in situ* hybridization: expression in P2 and midline first, followed by expression in the other pouches (fig. 11 in^21^). The midline *krt4*+ cells bear similarities to periderm: (1) they express some of the markers (krt4, zc1044) expressed by true peridermal cells; (2) they develop microridges similar to those in the periderm; (3) the *krt4*+ cell layer merges imperceptibly with peridermal cells invading from outside, suggesting compatible adhesion. They furthermore display ultrastructural features distinctive from the endodermal cells on which they rest. Expression of *krt4* (and its disputed homologues *krt8* and *zf*-*K8*) has been reported before in the pharynx of zebrafish^19,22,23^. Yet, none of the previous studies addresses the origin of these cells, nor how their domain expands, nor makes any suggestion regarding their periderm-like phenotype or association with the endoderm-derived epithelium. Our observations that the endoderm is covered by peridermal cells (in the pouches) and cells with a periderm-like phenotype (along the midline), generating two apposed, double-layered, epithelia in which then a lumen appears, fit with observations of Waterman & Kao^24^. These authors described the endoderm of the zebrafish pharynx as two-layered, becoming four-layered (a bilayer on each side of the lumen) in more advanced developmental stages. They also reported that superficial cells surrounding the lumen stain more intensely than the basal cells (visible on their Fig. 9). The superficial dark staining layer corresponds to the *krt4*+ and electron-dense superficial layer described here. Edwards^25^ already reported the invasion of the pharynx by ectodermal cells in the carp, a close relative of the zebrafish, and suggested it to be a feature common to all pouches. We agree with Edwards’ observations, but we differ from Edwards’ interpretation in that we distinguish periderm from ectoderm cells, and that invading cells do not reach the midline, but are joined instead by midline *krt4*+ cells. The situation in carp and zebrafish may be common for teleosts, as Miyake *et al*.^26^ describe an ectoderm-derived inner lining of the opercular cavity in cod (*Gadus morhua*) and Yamamoto *et al*.^27^ report the presence of flattened epithelial cells covering the hatching gland cells in the medaka pharynx (and see also Fig. 6). In contrast, Shone & Graham^28^ report only endoderm in the pouches of zebrafish, reaching out to the exterior, covering the inside of the opercular flap at 72 hpf. However, they used the endodermal marker *sox 17* only and did not investigate the number of cell layers. Our observation that the pouches are invaded by peridermal cells that subsequently constitute part of the permanent lining of the pharyngeal arches provides further evidence that the zebrafish periderm is more than just a transient embryonic feature^29^. It confirms that cells of the extra-embryonic enveloping layer (EVL, the source of the periderm) survive well into later embryonic stages or even juvenile life, as demonstrated previously for the epidermis^30,31^. While the periderm in zebrafish, by origin, is an extra-embryonic layer, periderm in mammals derives from ectoderm. Yet expression of some orthologous keratin genes, such as K8, appears to be shared^31,32^. That the midline *krt4*+ cells have an origin distinct from the skin periderm appears to be well established: (1) the cells are present prior to any periderm migrating into the pouches (as inferred from the cell tracing experiments); (2) while forming a continuous sheet, there is a sharp boundary between periderm cells and midline *krt4*+ cells, as revealed by double transgenic embryos; this lack of gradient strongly suggests a different origin; (3) while sharing several markers, they also differ in immunoreactivity when stained with the AE1/AE3 keratin antibody. The source of the midline *krt4*+ cells is intriguing. Initially, the cells form a cluster located in the midline region at the level of P1-P2, a region typically harboring *sox17*+ cells. The weak expression of *krt4* in the midline cells of early double transgenic (endoderm/periderm) embryos however precluded colocalization of *sox17*+ endodermal cells and midline *krt4*+ cells. If the midline *krt4*+ cells derive from the same source as the pharyngeal endoderm proper and adopt a periderm-like phenotype this could represent a case of co-option^33^. It could also explain why the cells turn on *sox17* expression at a later stage of embryonic development. From the area of origin, *krt4*+ cells could spread into the pharynx by migrating out of this region. Alternatively, *krt4* expressing cells could arise locally. Due to the deep internal position of the cells, dual photon microscopy could not rule out any of the two alternatives. Most evidence speaks against a local origin of the *krt4*+ midline cells. First, it is unlikely that the two apposed monolayers of endodermal cells itself upregulate *krt4* simply because *krt4*+ cells are present only where more than two cell layers are found along the midline, with *krt4*+ cells trapped between *sox17* expressing cells. If derived locally, *krt4*+ cells should issue from the division of endodermal cells perpendicular to their apicobasal axis, generating a basal cuboidal endodermal cell and an apically positioned flattened cell, instantaneously expressing *krt4*. On semithin sections, metaphases indicating such polarized division within the endodermal cells were not detected. Mitoses were rare along the midline once the two opposing endodermal monolayers were established, but more frequent in the distal parts of the endodermal pouches, where cell division likely contributes to lateral pouch outgrowth. Thus, the more likely explanation is that cells migrate from a source located at the level of P1-P2. That the midline cells are rich in Krt4 raises the question whether this keratin could play a role in such a migration. Intermediate filaments, and in particular keratins, are increasingly considered to play a key role in epithelial cell migration^34^. For example, appropriate recruitment of keratin intermediate filaments is required for the directed protrusive behavior of individual cells within the collectively migrating mesendoderm in *Xenopus*^35^.

A likely function of the midline *krt4*+ cells and the peridermal cells in the pouches is in lumen formation. Lumina in the pharynx and pouches arise in a non-confluent way but appear only where *krt4*-expressing cells are present. These cells may thus function in an analogous way as the mammalian periderm. In mammals, the periderm prevents premature adhesion between closely apposed epithelia and needs to be removed in order for epithelia to fuse, as in palatogenesis; reviewed in^36^. In the zebrafish pharynx and in the pouches, the presence of cells with a peridermal phenotype appears equally to be required to move apposed endodermal layers apart and to allow lumen formation. If, and to what extent, the midline *krt4*+ cells participate in the formation of pharyngeal derivatives is not known at present but is worthwhile investigating, considering the timing of their appearance. For example, the first thyroid follicle develops from the ventral midline epithelium of the pharynx at around 60 hpf^37^ and the thymus appears at 54 hpf^38^, i.e. right after the midline *krt4*+ cells have covered the endoderm. Likewise, whether the midline *krt4*+ cells exchange signals with the endoderm, and/or underlying mesenchyme and play a role in the development of ectomesenchymal derivatives, is currently unknown. The presence of long cell extensions reaching down to the basal lamina between endodermal cells is intriguing in this respect. While interactions between periderm and endoderm have not been reported before, signaling between ectoderm and endoderm is crucial for several developmental processes in zebrafish, such as for the development of gill filament buds^39,40^, or for epibranchial neurogenesis^41,42^. Ectodermally expressed Wnt4a signals to endodermal pouch epithelium and acts together with EphrinB signaling to stabilize mature bilayers in the pouch endoderm^43,44^. In amphibians, ectodermal taste buds are induced by endoderm^45^. In mice endoderm-ectoderm signaling has been reported during early jaw morphogenesis^46^. The thymus defect in the nude mouse has been attributed to an arrest of development of ectoderm in the third cleft, depriving the endoderm from “its normal inducing agent”^47^. Clearly, the potential role of the periderm and of the midline *krt4*+ cells in the development of pharyngeal derivatives needs to be investigated. Advantage can be taken from the powerful tools that have been recently developed to study ectoderm and periderm fate^20^ and that have also been used here.

The boundary between germ layers and the delimitation of endoderm has received most attention in relation to mouth formation and mouth opening. Using lineage tracing, we observed that peridermal, *krt4* expressing cells also enter the stomodeum, but only for a limited distance and after *krt4*+ cells have appeared along the midline at the level of P1-P2. Subsequently, the two populations meet and merge into an uninterrupted layer, indicating striking similarities in the events of mouth and pouch formation. These observations may further fuel debates on the homology of chordate mouth and gill slits, as e.g., recently revived for Amphioxus^48,49^. Once the mouth opens and the pouches open into gill slits, the oropharyngeal cavity is communicating with, and forms an extension of, the external environment. From a functional perspective, it is beneficial to have a pharynx that is lined by tissue adapted to interact with the aqueous environment, similar to what periderm in the skin does. From an evolutionary perspective, one would expect such a role to be fulfilled by invading ectoderm. For example, the capacity of tooth formation in the oropharynx has been proposed to be linked to an invasion of ectoderm^50^. It is thus somehow surprising that in zebrafish expansion of periderm into the oropharynx is arrested in the distal parts of the mouth and pouches, and that elsewhere endoderm has possibly been co-opted into a peridermal fate. That other (both basal and derived) teleost species display a two-layered pharyngeal epithelium early in development, with superficial flattened cells different from basal cuboidal cells, warrants investigations into the commonality of invasion and co-option in the development of the pharynx lining.

## Materials and Methods

### Transgenic zebrafish lines

Tg(*sox17:egfp*)^51^ and wildtype (AB line) zebrafish were obtained from the laboratory of R. Opitz (VUB, Brussels, Belgium). Tg(*krt4:gfp*) fish^19^ and Tg(*krt4:tomatoCAAX*) were a gift from M. Hammerschmidt^52^ (University of Köln, Germany). The Tg(*sox17:egfp*) was crossed to the Tg(*krt4:tomatoCAAX*) line. Et(Gal4-VP16)^zc1044A^;Tg(UAS-E1b:nsfB-mCherry)^c264^, abbreviated as GET-periderm line (GET, Gal4 enhancer trap)^20^ was crossed to the Tg(*krt4:gfp*) line. Adult fish were maintained and spawned according to^53^. Embryos were raised in egg water at 28.5 °C and staged according to^15^.

### CDCFDA labeling

For cell lineage tracing, WT embryos were soaked in CDCFDA [5-(and-6)-carboxy-2′,7′-dichlorofluorescein diacetate, succinimidyl ester, mixed isomers] (cat. No.: 22026, AAT Bioquest, Inc.). This molecule, an isomer of the molecule used by Shone & Graham^28^ is taken up by any cell that is in direct contact with the agent and does not diffuse further. It is thus limited to cells exposed to the aqueous environment. CDCFDA was dissolved to 50 mM in anhydrous DMSO (dimethylsulfoxide) and stored at −20 °C. A working concentration of 250 µM CDCFDA was dissolved in egg water and embryos were soaked for 4 hours starting at 6 hpf, and then every two hours until 18 hpf, as well as at 26 hpf. After 4 hours in CDCFDA staining, the embryos were rinsed in fresh egg water and kept in petri dishes with fresh egg water until sacrifice. Embryos were fixed in 4% PFA and processed for glycol methacrylate (GMA) embedding.

### Periderm removal

Periderm was removed from Tg(*krt4:gfp*) fish by soaking Tg(*krt4:gfp*) embryos at 26 hpf for 15 minutes in 12.5 mM EDTA dissolved in Ca^2+^ and Mg^+^ free Tyrode solution. Afterwards, they were washed several times and maintained in regular Tyrode solution. Embryos were sacrificed at various intervals and processed for GMA embedding.

### BrdU incorporation

Proliferation at the level of the pharynx was examined through BrdU (5-bromo-2′-deoxyuridine) administration followed by immunostaining on whole-mount embryos. Embryos were treated with BrdU at 36 hpf according to the protocol described in^54^, sacrificed immediately after BrdU administration and fixed in 4% PFA for GMA embedding.

### Immunohistochemistry

Immunohistochemistry on whole-mount embryos was performed as described in^54^ using as primary antibodies an anti-BrdU (mouse, BD Biosciences, 1:100), a Zn-8 (mouse, ZIRC, 1:100) or a pan-cytokeratin (AE1/AE3) (mouse, Santa Cruz, 1:200) and as secondary antibody an Alexa Fluor® 594 (goat anti-mouse IgG, Abcam, 1:200).

### Histology & TEM

All animals were sacrificed by an overdose of 1% ethyl 3-aminobenzoate methansulfonate (MS-222) (E10521-10G, Sigma Aldrich) and fixed overnight in 4% paraformaldehyde (PFA in 1xPBS, pH 7.2) at 4 °C. The embryos were processed for GMA embedding after immunohistochemistry, vital staining or without pretreatment (transgenic zebrafish), and serially sectioned at 3 µm. After proper drying of the sections, some sections were counterstained with DAPI for nuclear staining. Fluorescent signals (GFP, DyLight, or CDCFDA) were visualized by placing a drop of 1xPBS per slide, and coverslipping. After analysis, sections were stained with toluidine blue. For high resolution histology and transmission electron microscopy (TEM), embryos were fixed in a mixture of paraformaldehyde and glutaraldehyde, embedded in epon, serially sectioned at 1 µm^55^, and stained with toluidine blue. Ultrathin sections were prepared on an ultratome, contrasted with uranyl acetate and lead citrate, and observed under a Jeol JEM 1010 transmission electron microscope (Jeol Ltd., Tokyo, Japan) operating at 60 kV. Microphotographs were taken with a Veleta camera (Emsis, Muenster, Germany).

Toluidine blue stained sections from GMA or epon embedded material of *Anguilla anguilla*, *Oncorhynchus tshawytscha*, and *Hemichromis bimaculatus*, are part of the collections maintained in the Research Group Evolutionary Developmental Biology (Ghent University).

### Dual photon microscopy

The Tg(*krt4*:*gfp*) fish were dechorionated and were immobilized at 30 hpf in agar diluted in egg water to a concentration of 0.1%. The embryo was positioned to obtain a dorso-lateral view and a sequence was recorded on a Zeiss LSM 780 confocal microscope of 32 Z-stacks every 20 minutes for a total of 18 hours. The pictures and video were extracted from records using Zeiss Axio Imager Z1 (www.zeiss.com) and Image J (version 2.0.0-rc-29, plus Bio-Formats 5.1.0 in Fiji, general public license).

### Observations and microphotography

Observations of epon or GMA sections were done on a Zeiss Axio Imager Z1 (www.zeiss.com). Photomicrographs were taken with an MRC camera and processed using ZEN software (Zeiss, www.zeiss.com). Computer-generated images were processed for color balance, contrast and brightness only, and applied to all parts of the figures equally.

### Ethical statement

Animal care, experimentation and sacrifice complied with European Directive 2010/63/EU of 22 September, 2010. The experimental protocol and all animal procedures used in this study were approved by Ghent University (laboratory permit number LA1400452).

## Supplementary information

Supplementary Information

Movie

## Data Availability

All sections used for this study are kept in the slide collection of the Research Group ‘Evolutionary Developmental Biology’ at the Biology Department of Ghent University, and are available for inspection upon request.
