# Supplementary material for: Periderm invasion contributes to epithelial formation in the teleost pharynx
Source: Sci Rep. 2019 Jul 12;9:10082. doi: 10.1038/s41598-019-46040-y (PMC6626026; doi:10.1038/s41598-019-46040-y)
Supplement: Supplementary file 1 — Supplementary Information [file 41598_2019_46040_MOESM1_ESM.pdf]

Supplementary data

Periderm invasion contributes to epithelial formation in the teleost pharynx

Joana Teixeira Rosa[a,b,1], Veronika Oralová[a,c,1], Daria Larionova[a], G.T. Eisenhoffer[d], P. Eckhard Witten[a], and Ann Huysseune[a*]

Author affiliations:

[a]Research Group Evolutionary Developmental Biology, Biology Department, Ghent University. K.L. Ledeganckstraat 35, B-9000 Gent, Belgium

[b]Current address: Comparative, adaptive and functional skeletal biology (BIOSKEL), Centre of Marine Sciences (CCMAR), Building 7, University of Algarve, Campus Gambelas, 8005-139 Faro, Portugal

[c]Current address: Institute of Animal Physiology and Genetics, v.v.i., Czech Academy of Sciences, Veveri 97, 602 00, Brno, Czech Republic

[d]Department of Genetics, MD Anderson Cancer Center, 1515 Holcombe Blvd., Unit 1010, The University of Texas MD Anderson Cancer Center, Houston, Texas 77030, USA

[1] J.T.R. and V.O. contributed equally to this work.

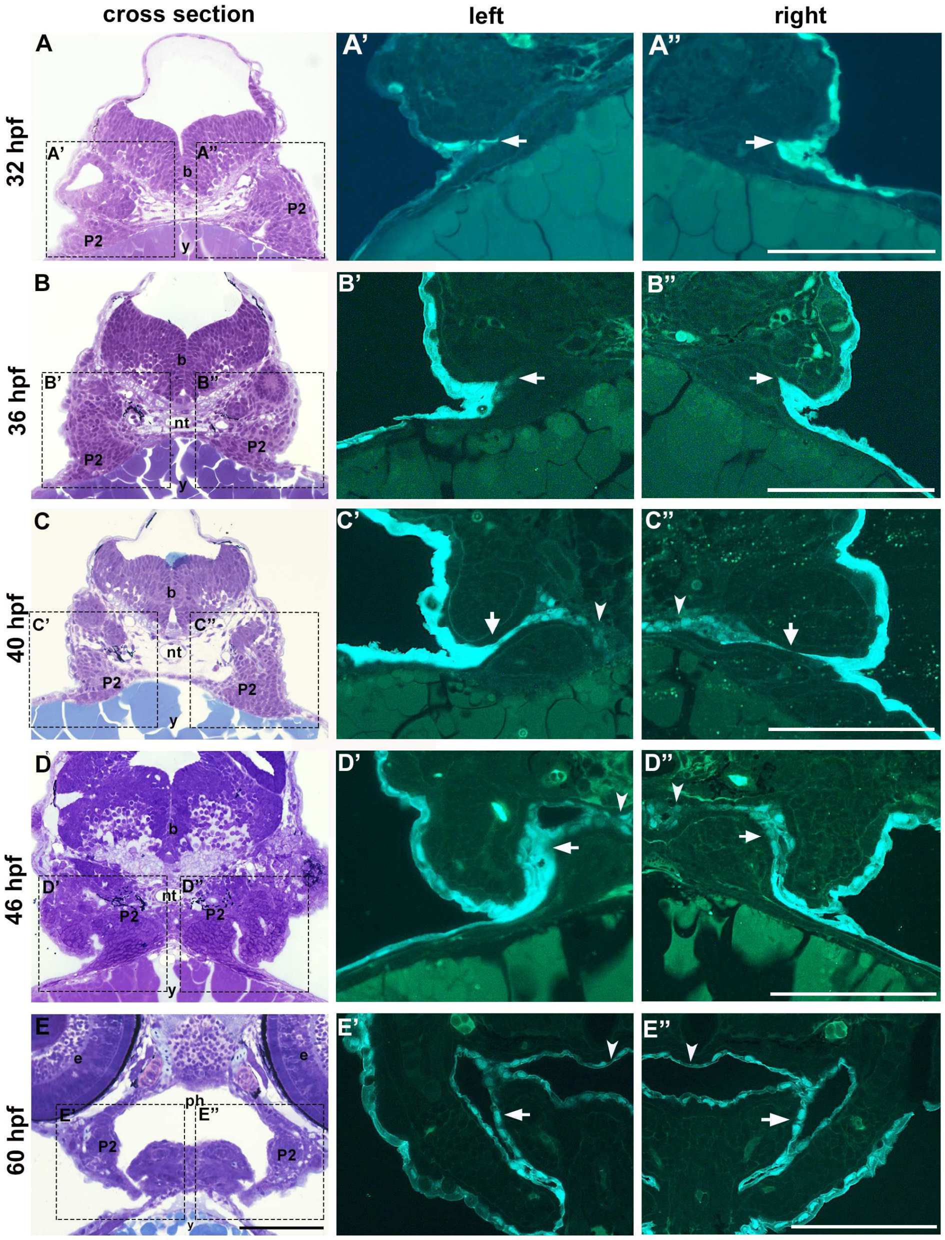

Figure S1. Invasion of peridermal cells through P2 and connection to midline *krt4*+ cells. (A, B, C, D, E) Toluidine blue staining of 32, 36, 40, 46 and 60 hpf zebrafish, resp., showing the area of pouch 2 (P2) in cross sections. **(A', B', C', D', E')** Cross sections of the left and **(A'', B'', C'', D'', E'')** right sides of Tg(*krt4:gfp*) embryos with *krt4*+ peridermal cells first covering the outside of the embryo only, then squeezing in between endodermal cells of P2 (arrows), and making contact with midline *krt4*+ cells (arrowheads). Eventually (E'-E''), a layer of *krt4*+ epithelial cells lines the roof (arrowheads) and floor of the pharynx continuing into the lining of P2 (arrows). b, brain; e, eye; nt, notochord; ph, pharynx; P2, pouch 2; y, yolk; Scale bars = 100 μm.

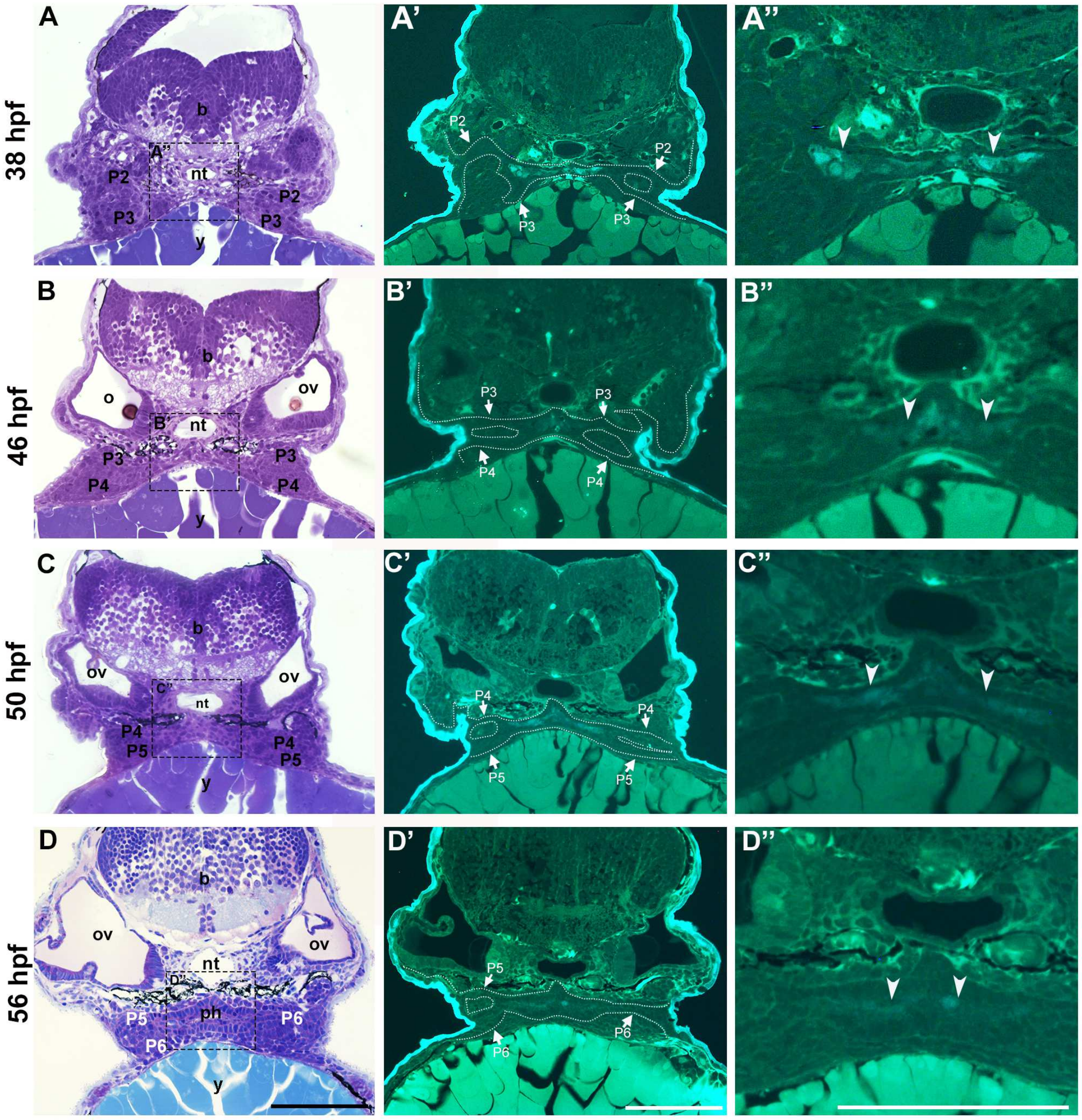

Figure S2. Expansion of midline *krt4*+ cells posteriorly. (A, B, C, D) Toluidine blue cross sections of 38, 46, 50 and 56 hpf zebrafish, resp., at the level of the different pouches. **(A', B', C', D')** Cross sections of Tg(*krt4:*gpf) embryos. *krt4*+ peridermal cells cover the outer surface of the zebrafish, and weaker *krt4*+ cells are found along the midline, but *krt4*+ cells remain as yet excluded from the pouches proper. **(A'', B'', C'', D'')** Magnification of the midline area with *krt4*+ cells marked by arrowheads. b, brain; nt, notochord; ov, otic vesicle; ph, pharynx; P2, pouch2; P3, pouch 3; P4, pouch 4, P5, pouch 5; P6; pouch 6. Scale bars = 100 µm.

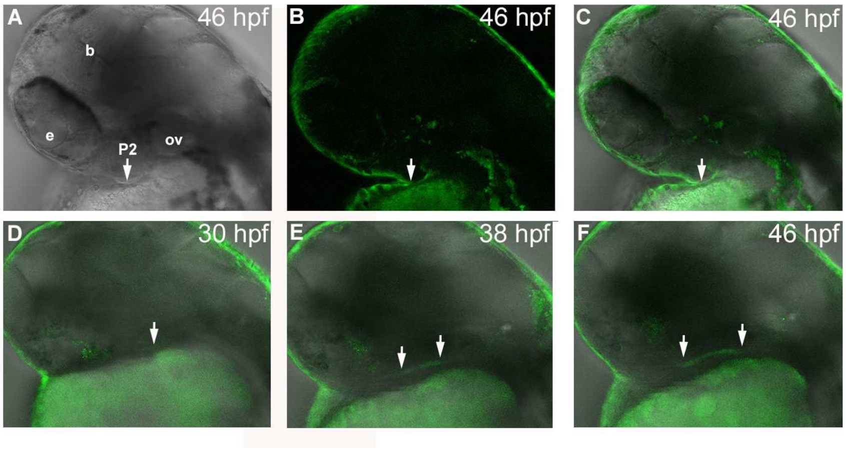

Figure S3. Dual photon recording. Still images of dual photon recording of a Tg*(krt4:gfp)* zebrafish at 46hpf at the level of P2 in brightfield (BF) **(A)**, with P2 marked (arrow). **(B)** Corresponding image in GFP canal and **(C)** GFP + BF canal with labeled P2 in green. **(D)-(F)** Still images from successive developmental stages showing progression of midline *krt4+* cells (arrows).

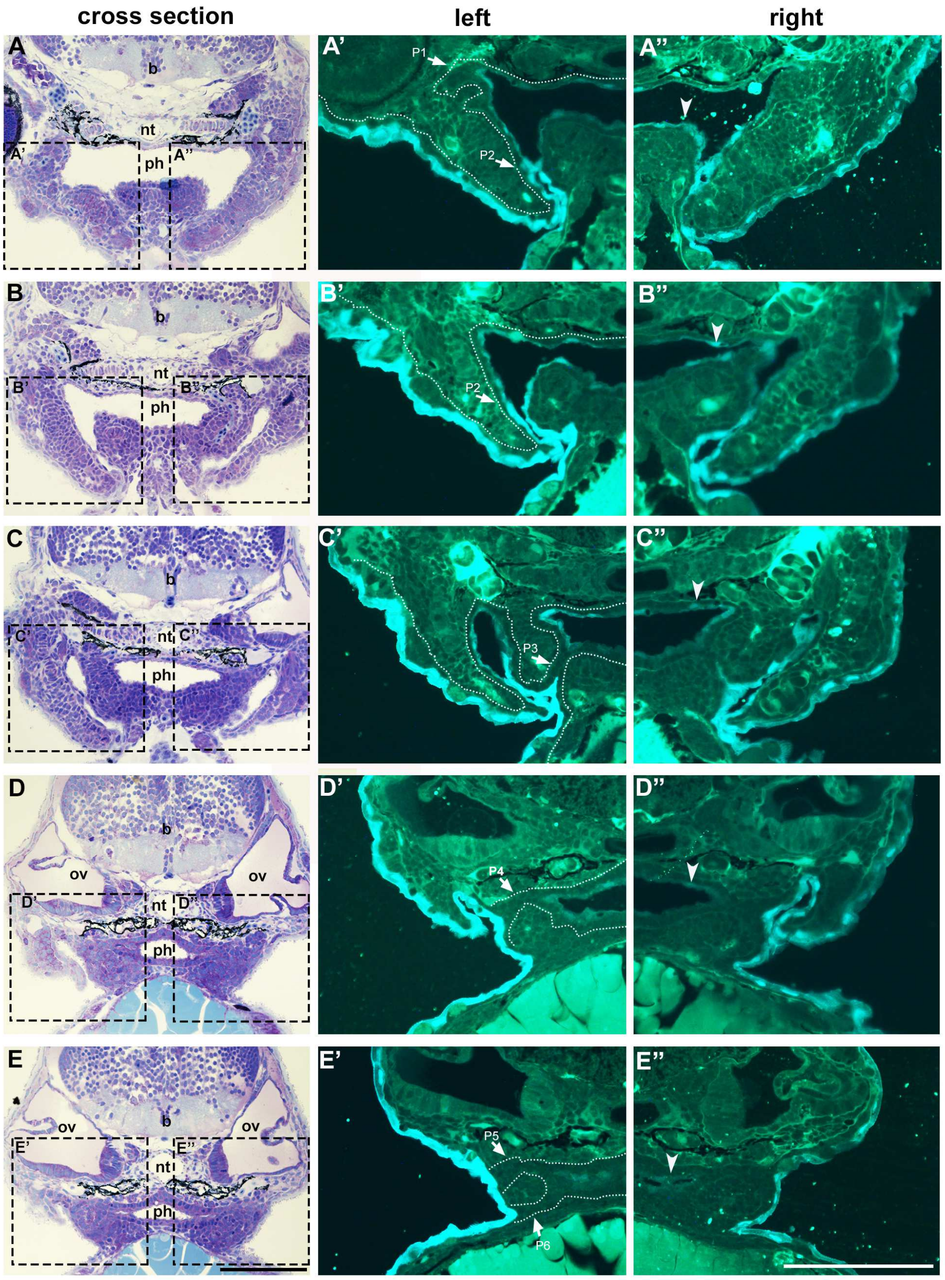

Figure S4. Late invasion of pouches 3 to 6 by periderm. (A – E) Toluidine blue stained cross sections of 56 hpf zebrafish with focus on the different pouches from anterior to posterior (P1 to P6). **(A'- E')** Cross section of left side with pouches labeled and **(A''- E'')** right side. **(A'-B'')** *krt4*+ cells cover the external surface of the embryo and have entered via P2 (arrows). Midline *krt4*+ cells cover the roof and floor of the pharynx lumen in the midline (arrowheads). **(C', C'')** At the level of P3, *krt4*+ cells have started to invade the pouch from outside (arrow). **(D',D'',E',E'')** *krt4*+ cells cover the midline (arrowhead) and the outer surface of the embryo, but are still excluded from the more posterior pouches (P4, P5 and P6). b, brain; nt, notochord; ph, pharynx; ov, otic vesicle; P1, pouch 1; P2, pouch 2; P3, pouch 3; P4, pouch 4; P5, pouch 5; P6, pouch 6. Scale bars = 100 μm.

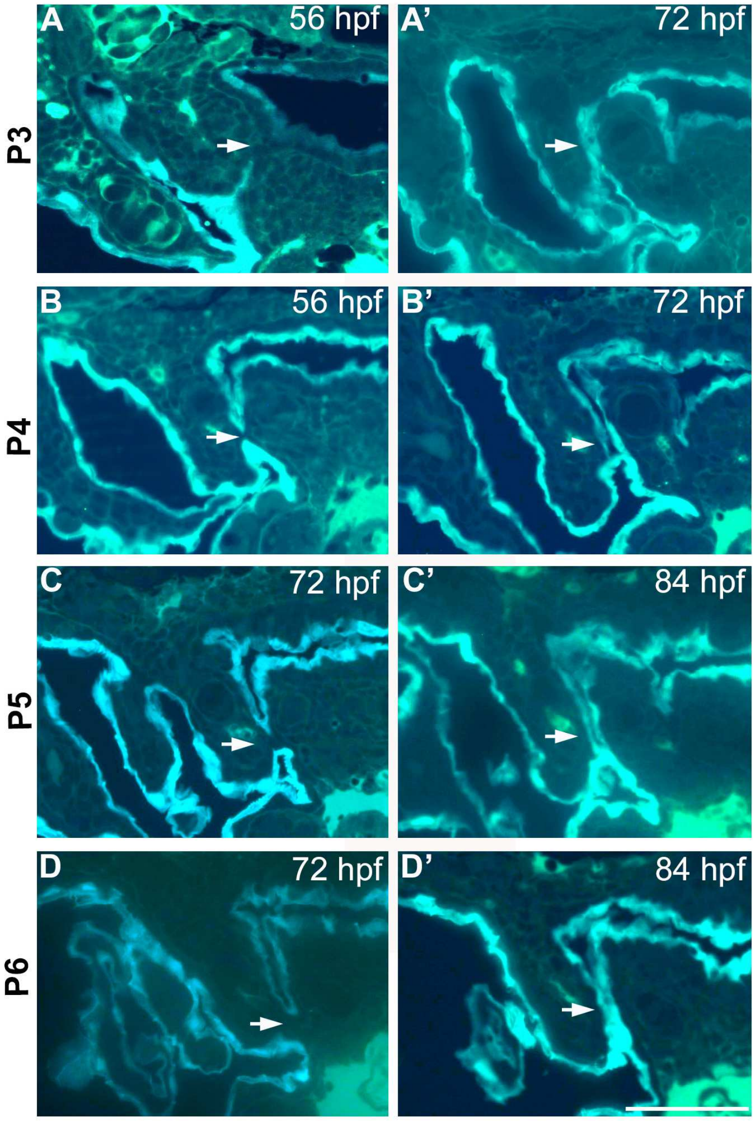

Figure S5. Expansion of midline *krt4*+ cells into pouches 3-6. **(A, B, C, D)** Cross sections of Tg(*krt4:gfp*) embryos with focus on the migration of peridermal cells via pouches P3, P4, P5 and P6, respectively, before they are met by midline *krt4*+ cells (arrows). **(A', B', C', D')** Detail of pouches P3-P6 after having been covered by *krt4*+ cells and lumen formation is almost completed (arrows), with times indicated for each pouch. Scale bar = 50 µm.

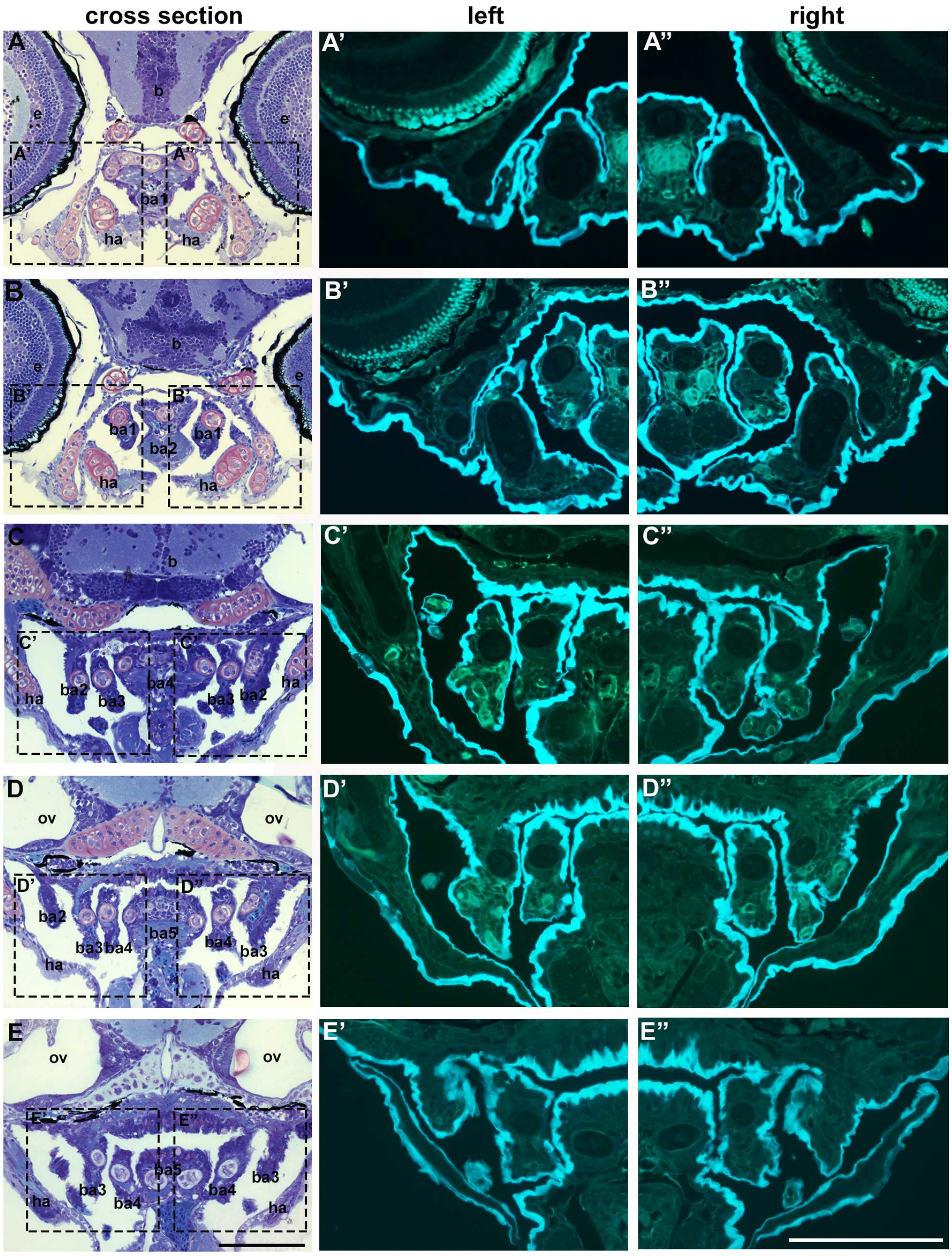

Fig. S6. Opening of pouches 2 to 6. (A - E) Toluidine blue stained cross sections of zebrafish at 5dpf from anterior to posterior focusing on the now opened pouches. **(A'- E')** Magnifications of right and **(A''- E'')** left side. *krt4+* cells cover the complete pharynx and all branchial arches from ba1 to ba5. b, brain; ba, branchial arch; e, eye; ha, hyoid arch; ph, pharynx; ov, otic vesicle. Scale bars = 100 µm.

Supplementary Movie

Dual photon recording of a Tg*(krt4:gfp)* zebrafish between 30 and 48hpf, dorso-lateral view of the head, showing invasion of periderm cells via P2. For still pictures and explanations, see Fig. S3A-C. Anterior to the right, ventral to the top.
